# Impact of Endpoint Delay on the Efficiency of Multi Arm Multi Stage Trials

**DOI:** 10.1002/sim.70245

**Published:** 2025-09-15

**Authors:** Aritra Mukherjee, James M. S. Wason

**Affiliations:** ^1^ Population Health Sciences Institute Newcastle University Newcastle Upon Tyne UK

**Keywords:** adaptive design, efficiency, multi‐arm multi‐stage (MAMS), outcome delay

## Abstract

Multi‐arm multi‐stage (MAMS) is an efficient class of trial designs that helps to assess multiple treatment strategies at the same time using an adaptive design. These designs can substantially reduce the average number of samples required compared to an equivalent single stage multi‐arm trial. However, if patient recruitment is continued while we await treatment outcomes, a long‐term primary outcome leads to a number of ‘pipeline’ patients getting recruited in the trial, who do not benefit from the early termination of a futile arm. This study focuses on quantifying the efficiency loss a MAMS design undergoes, in terms of the expected sample size (ESS), because of outcome delay. We first estimate the number of ‘pipeline’ patients (recruited during the interim analysis (IA) while awaiting outcome data) analytically through different recruitment models, given the total recruitment time. We then compute the ESS accounting for delay and assess the Efficiency Loss (EL). The results indicate that more than 50% of the expected efficiency gain is typically lost due to delay when the delay is more than 1/3rd of the total recruitment length. Although the number of stages have little influence on the efficiency loss, the timing of the IA can impact the efficiency of MAMS designs with delayed outcomes; in particular, conducting the IAs earlier than an equally‐spaced design can be harmful for the design. Finally, we conclude that, in order to gain maximum benefit of MAMS in terms of a reduced sample size in multi‐arm trials, the outcome delay should be less than a third of the total recruitment length.

## 1 Introduction

In recent years multi‐arm multi‐stage (MAMS) designs have gained much popularity due to their ability to compare multiple treatment arms simultaneously. As the name suggests, in a MAMS design, multiple treatment arms can be compared against a single control arm, with the aim of finding a single or multiple effective treatment arms [1, 2, 3]. This class of design helps to focus enrollment of patients more on treatments which are showing good efficacy, thereby simultaneously restricting the number of patients recruited to ineffective treatment arms. MAMS designs also allow for further adaptations to be introduced in the trial, for example, response adaptive randomisations or sample size re‐estimation based on the trial's objectives. The adaptations implemented in a MAMS design are typically predetermined to control type I error and power at specified levels. The stopping rules can also be ‘simultaneous’ or ‘separate’ in nature, that is, the trial can be stopped if one effective treatment is identified, or multiple effective treatments are identified. There are several sub‐types of MAMS design, including group‐sequential and drop‐the‐loser (DTL) approaches [4]. The biggest benefit that a MAMS trial provides is arguably the ability to answer multiple research questions simultaneously under a single trial protocol, rather than answering them sequentially or via a series of separate trials [5]. This reduces the time to identify an effective treatment, helps reduce the financial burden, while also adding to patient benefits.

However, recent studies have been exploring the impact of delay in assessing the primary outcome on the efficiency of several adaptive designs. Wason et al. has pointed out the harmful impact of delayed outcome on the efficiency of adaptive designs [6]. Mukherjee et al. discussed the problem in the context of neurosurgery trials [7]. Mukherjee et al. also has more formally explored the efficiency loss due to delay for a single‐arm two stage design [8] as well as a multi‐stage group sequential design (GSD) [9]. It has been observed that in both of these designs, a trial's efficiency can suffer greatly if the time to observe the primary outcome is relatively large compared to the total recruitment time. A common inference drawn from these two studies is that the expected sample size (ESS) is particularly sensitive to a delayed outcome. Similarly to the aforementioned designs, efficiency of MAMS designs has also been assessed based on their ESS [2, 10]. Thus, MAMS may have similar disadvantages in terms of an increased ESS as a result of a long‐term primary outcome. A previous study by Granholm et al. explored the impact of lagged outcome data on different adaptations for MAMS trials [11] with a Bayesian framework. Simulation studies were used to explore changes in statistical properties. In this article, we provide further insight into this issue by proposing analytical formulae for the efficiency loss (EL) due to endpoint delay in MAMS designs. Our objective is to observe the impact of delayed outcomes on MAMS within a frequentist viewpoint. Particularly, we have focused on a group‐sequential MAMS design with separate stopping rules due to its common usage. The impact of delayed outcome is assessed based on the number of pipeline patients recruited in the trial while awaiting treatment outcome data. The participants who are recruited while treatment outcomes are awaited during the interim analysis are termed as pipeline patients. We assume that, recruitment is not paused during the interim analysis. The method to obtain these estimates of pipeline participants is described in detail in the Methods section, following a brief introduction to the design methodology and notation. The efficiency is measured through the efficiency loss (EL), given as the relative proportion of expected efficiency gain lost due to the increase in ESS from delay.

## 2 Methods

### 2.1 Setting and Notation

We adopt a similar setting to Magirr et al. [12], with the following notation. Let us consider that we want to test the efficacy of K experimental treatment arms against a control arm in a J‐staged MAMS. Let, $X_{ijk}$ denote the treatment outcome for the $i^{th}$ patient in the $k^{th}$ treatment arm at stage $j,i=1,2,\ldots ,n_{j}k$; j=1,2,…,J;k=0,1,2,…,K, where $n_{jk}$ denotes the sample size for the $k^{th}$ arm at stage j. Further, let $\mu_{k},k=1,2,\ldots ,K$ be the mean response on the experimental treatments and $\mu_{0}$ be the mean response on the control, that is,

$$X_{ijk}∼N\left(\mu_{k},\sigma_{k}^{2}\right);k=0,1,2,\ldots ,\;K$$

Then, the hypotheses under test are given by, $H_{k}:\delta_{k}=\mu_{k}-\mu_{0}\leq 0;k=1,2,\ldots ,K.$

Let us assume that the $j^{th}$ interim analysis is planned after $n_{j}$ participants have had their outcome assessed. Thus, $n_{j}=\sum_{1}^{j}\sum_{0}^{K}n_{jk}\theta_{jk}$ and the maximum sample size required for a MAMS is denoted by $n_{\max}=\sum_{1}^{J}\sum_{0}^{K}n_{jk}$. Here, $\theta_{jk}$ denotes the indicator variable for representing whether the $k^{th}$ treatment arm is present in the $j^{th}$ analysis. The exact expression for $\theta_{jk}$ can be obtained from Equation (1).

After each stage j, we compute test statistics $Z=Z_{jk},\;j=1,2,\ldots ,\;J;\;\;k=0,\;1,2,\ldots ,K$ comparing each experimental arm to control, defined as

$$Z_{jk}=\frac{\frac{1}{n_{jk}}\sum_{i=1}^{n_{jk}}X_{ijk}-\frac{1}{n_{0k}}\sum_{i=1}^{n_{0k}}X_{ij0}}{\sqrt{\left(\frac{\sigma_{k}^{2}}{n_{jk}}+\frac{\sigma_{0}^{2}}{n_{j0}}\right)}}={\hat{\tau }}_{jk}I_{jk}^{1/2}$$

where, ${\hat{\tau }}_{jk}=\left(\frac{1}{n_{jk}}\sum_{i=1}^{n_{jk}}X_{ijk}-\frac{1}{n_{0k}}\sum_{i=1}^{n_{0k}}X_{ij0}\right)$ is the observed treatment effect and $I_{jk}={\left(\sqrt{\left(\frac{\sigma_{k}^{2}}{n_{jk}}+\frac{\sigma_{0}^{2}}{n_{j0}}\right)}\right)}^{-1}$ is the information for treatment k in stage j.

Let us denote by $\tau =\left(\tau_{1},\tau_{2},\ldots ,\;\tau_{K}\right)$, the vector of the true treatment effects. Then, the mean of $Z_{jk}$ and covariance between $Z_{jk}$ and $Z_{j^{\prime }k^{\prime }}$ are given as

$$\begin{array}{ll} & E(Z_{jk})=\tau_{k}I_{jk}^{1/2}=\frac{\tau_{k}}{\sqrt{\left(\frac{\sigma_{k}^{2}}{n_{jk}}+\frac{\sigma_{0}^{2}}{n_{j0}}\right)}}\\ & cov(Z_{jk},Z_{j^{\prime }k^{\prime }})=\left\{\begin{array}{ll} 1,\; & k=k^{\prime },j=j^{\prime }\\ I_{jk}I_{j^{\prime }k^{\prime }}\left(\frac{\sigma_{k}^{2}}{n_{jk}}+\frac{\sigma_{0}^{2}}{n_{j0}}\right),\; & k=k^{\prime },j<j^{\prime }\\ I_{jk}I_{j^{\prime }k^{\prime }}\frac{\sigma_{0}^{2}}{n_{j0}},\; & k\neq k^{\prime },j<j^{\prime } \end{array}\right.\\ & or,cov(Z_{jk},Z_{j^{\prime }k^{\prime }})=\left\{\begin{array}{ll} 1,\; & k=k^{\prime },j=j^{\prime }\\ \frac{\sqrt{\left(\frac{\sigma_{k}^{2}}{n_{jk}}+\frac{\sigma_{0}^{2}}{n_{j0}}\right)}}{\sqrt{\left(\frac{\sigma_{k^{\prime }}^{2}}{n_{j^{\prime }k^{\prime }}}+\frac{\sigma_{0}^{2}}{n_{j^{\prime }0}}\right)}},\; & k=k^{\prime },j<j^{\prime }\\ \frac{\frac{\sigma_{0}^{2}}{n_{j0}}}{\sqrt{\left(\frac{\sigma_{k}^{2}}{n_{jk}}+\frac{\sigma_{0}^{2}}{n_{j0}}\right)\left(\frac{\sigma_{k^{\prime }}^{2}}{n_{j^{\prime }k^{\prime }}}+\frac{\sigma_{0}^{2}}{n_{j^{\prime }0}}\right)}},\; & k\neq k^{\prime },j<j^{\prime } \end{array}\right. \end{array}$$

In a group‐sequential MAMS trial, stopping boundaries are determined like in a two‐arm group‐sequential trial. These stopping boundaries determine what happens to each experimental arm at interim analyses. We define the stopping boundaries as (e,f), where, $e=(e_{1},e_{2},\;\ldots ,\;e_{J})$ represents efficacy stopping boundaries and $f=(f_{1},f_{2},\ldots ,\;f_{J})$ denotes futility stopping boundaries for J stages. Then, following [13], the formal conduct of the trial is expressed using two vectors, $\psi =\;{\left(\psi_{1},\ldots ,\psi_{K}\right)}^{T}$ and $\omega ={\left(\omega_{1},\ldots \;,\omega_{K}\right)}^{T}$, as follows

1. Set $\psi \;=\omega =0_{K}$ and j=1.
2. Conduct stage j of the trial, and compute the $Z_{jk}$ for all k present in stage j.
3. For the $k^{th}$ treatment arm,
  - if $Z_{jk}>e_{j}$, treatment k is considered efficacious. Here, denote $\psi_{k}=1$ and $\omega_{k}=j$ and set $Z_{lk}=\infty$ for l=j+1,…,J.
  - if $Z_{jk}\leq f_{j}$, the $k^{th}$ treatment is considered futile. Here, denote $\psi_{k}=0$ and $\omega_{k}=j$ and set $Z_{lk}=-\infty$ for l=j+1,…,J.
  - the trial is continued to stage j+1, if $f_{j}<Z_{jk}<e_{j}$.
4. Repeat steps 2 and 3 till a decision has been made for all K null hypotheses.

For the final analysis, we consider, $f_{J}=e_{J}$, ensuring the trial is terminated at stage J. Thus, for $k=1,2,\ldots ,K,\psi_{k}$ can either be 1 or 0 depending on whether $H_{k}$ is rejected or not and $\omega_{k}$ can take any value from 1 to J depending on at which stage the decision is made for the $k^{th}$ treatment. The realised sample size for any MAMS is highly dependent on the value of ω: we denote it by n(ω).

If (Ω,Ψ) denotes the sample space for all possible combination of values of (ω,ψ) for a MAMS with J stages and K arms, then the expected sample size (ESS) of a MAMS can be given as:

$$ESS(\tau ,e,f)=\underset{\left(\omega ,\psi )\epsilon (\Omega ,\Psi \right)}{\sum }n(\omega )P(\omega ,\psi |\tau ,e,f)$$

where P(ω,ψ|τ,e,f) denotes the probability of a particular value of (ω,ψ)ϵ(Ω,Ψ) given the stopping boundaries (e,f) and treatment effect τ.

P(ω,ψ|τ,e,f) can be derived using a JK‐dimensional integral on the multivariate normal distribution of Z (see Grayling et al. [13] for more details).

### 2.2 Efficiency Metrics

The efficiency a MAMS design provides can be quantified through how much it reduces the ESS as compared to an equivalent multi‐armed trial with no interim analyses. Specifically, keeping the number of arms fixed at K, the efficiency gained as a result of introducing J interim analyses, can be compared against a K‐armed single stage trial.

The expected efficiency gain (EG) from using a MAMS instead of a corresponding K‐armed single‐stage design can be calculated as

$$EG\left(\tau ,e,f\right)=\frac{n_{MA}-ESS(\tau ,e,f)}{n_{MA}}$$

where $n_{MA}$ is the required sample size for the K‐arm single‐stage design.

However, if there is delay in observing the primary treatment outcome and recruitment is continued while we wait for the treatment outcome to be available, there will be so‐called ‘pipeline’ patients recruited in the trial who do not contribute to the interim analysis because their outcome is not yet observed. Let us denote by $t_{\max}$, the time to recruit all $n_{\max}$ patients and $m_{0}$ be the time to observe the primary outcome. Let, $n_{\text{delay}}(\omega )$ denote the realised sample size including the pipeline participants accumulated due to a delayed outcome.

Then $ESS_{\text{delay}}(\tau ,e,f,m_{0},t_{\max})$ should be defined using $n_{\text{delay}}(\omega )$ in place of n(ω) in the expression of ESS given in the above section. This accounts for the extra pipeline patients, recruited in the trial at each interim analysis.

Thus, the ‘true’ efficiency gained (EG) compared to a single‐stage design in the presence of outcome delay can be measured as

$$EG_{\text{delay}}(\tau ,e,f,m_{0},t_{\max})=\frac{n_{MA}-\;ESS_{\text{delay}}(\tau ,e,f,m_{0},t_{\max})}{n_{MA}}$$

We will then quantify the efficiency loss (EL) due to outcome delay as the percentage change in the EG when considering delay in comparison to not considering delay. That is

$$\begin{array}{ll} & \;EL\left(\tau ,e,f,m_{0},t_{\max}\right)\\ & =100\left(\frac{EG(\tau ,e,f)-EG_{\text{delay}}(\tau ,e,f,m_{0},t_{\max})}{EG(\tau ,e,f)}\right) \end{array}$$

Note that the value of the EL is typically expected to be less than or equal to 100. However, it is possible to observe a value greater than 100 if the delay makes the MAMS less efficient than a multi‐arm trial.

An EL value of 100% indicates that the expected efficiency gain is completely lost due to delay, that is, the MAMS design effectively have the same expected sample size as a single stage multi‐arm trial. Whereas, if the EL is more than a 100% this implies, the MAMS design in reality recruits more patients than a K‐armed single stage design. Therefore effectively losing all the efficiency gain and increasing the loss further due to additional pipeline participants. The maximum EL will be observed if the MAMS recruits the maximum sample size $\left(n_{\max}\right)$, for a sufficiently large delay length.

### 2.3 Computing the Number of Pipeline Participants

Similar to our previous work, we consider two sub‐cases for estimating the number of pipeline participants at a given interim analysis. We also consider time to be a discrete variable and the unit of time to be months, though the results can be readily generalised for other units of time given all parameters are defined in the same units.

First, we define the following terms for ease of computing the number of pipelines.

For a given (ω,ψ), let us denote the realised number of stages as $j_{\max}=max\left\{\omega_{1},\omega_{2},\ldots ,\omega_{K}\right\}$ and the indicator variable for representing whether the $k^{th}$ treatment arm is present in the $j^{th}$ analysis as

(1) $$\begin{array}{l} \theta_{jk}=\left\{\begin{array}{l} 1;\;\;\omega_{k}\leq j\\ 0;\;\;otherwise \end{array}\right.k=1,\ldots ,K;j=1,2,\ldots ,j_{\max} \end{array}$$

Typically, for $k=0,\;\theta_{jk}=1\;\forall \;j=1,2,\ldots ,j_{\max}$ as the control arm is present in all the interim analysis. Hence, for a particular interim, we can obtain the number of treatment arms present in that interim as $\sum_{k=0}^{K}\theta_{jk}$.

**FIGURE 1:**
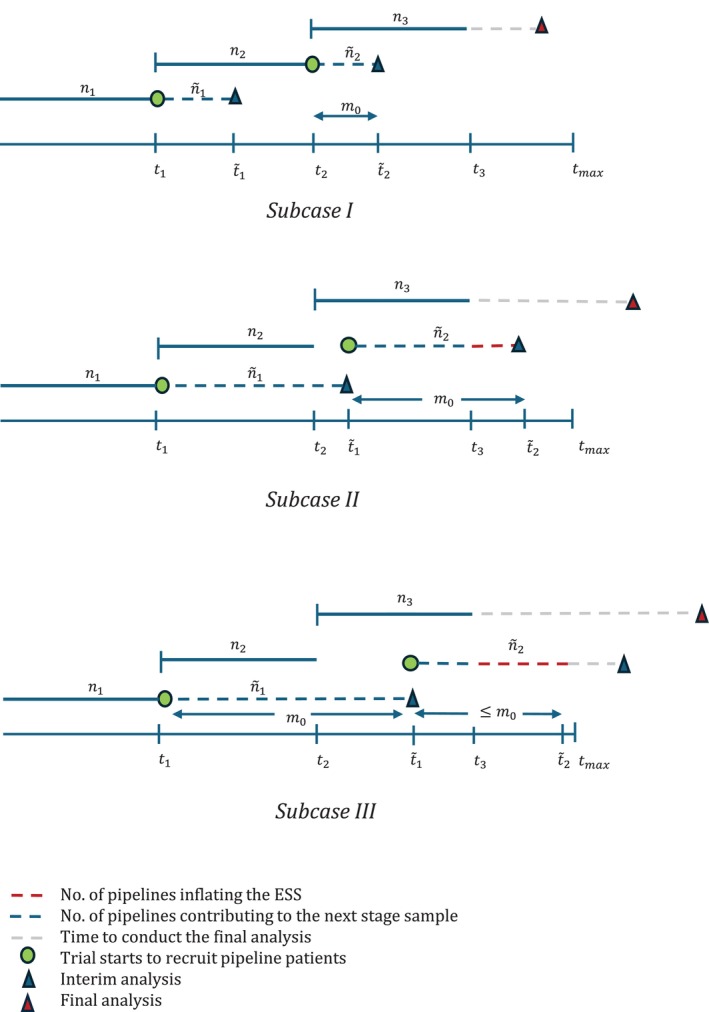
Different case scenarios as described in Equation (2) for different delay lengths for a 3 stage K‐arm trial.

Let us assume, it takes $t_{j}$ units of time to recruit all $n_{j}$ patients at stage j and let ${\tilde{t}}_{j}$ denote the time the $j^{th}$ interim analysis occurs at. Now, in most of the cases where treatment outcomes are immediately available after patient recruitment, $t_{j}={\tilde{t}}_{j}$. However, in presence of delay, these two can differ significantly. The value of ${\tilde{t}}_{j}$, in presence of delay, is highly dependent on ${\tilde{t}}_{j-1}$ (Figure 1).

In general,

(2) $$\begin{array}{l} {\tilde{t}}_{j}=\left\{\begin{array}{ll} t_{j}+m_{0},\; & {\tilde{t}}_{j-1}\leq t_{j}\\ {\tilde{t}}_{j-1}+m_{0},\; & t_{j-1}>t_{j}\;and\;{\tilde{t}}_{j-1}+m_{0}<t_{\max}\\ t_{\max},\; & t_{j-1}>t_{j}\;and\;{\tilde{t}}_{j-1}+m_{0}\geq t_{\max} \end{array}\right. \end{array}$$

If we assume that recruitment is not paused during the interim analyses, then there will be pipeline participants recruited in the trial at every interim while we await treatment outcome data. Let us denote, the number of pipeline participants recruited at stage j as ${\tilde{n}}_{j},\;j=1,2,\ldots ,\max\left\{j_{\max},J-1\right\}$ (there will be no pipeline participants at the final analysis of the trial). In order to find this ${\tilde{n}}_{j}$, we need an estimate of $t_{j}$ which depends on the recruitment rate of the trial. We will consider two recruitment models in Sections 2.3.1 and 2.3.2 respectively.

Along with the recruitment pattern, ${\tilde{n}}_{j}$ also depends on the number of treatment arms present in the $j^{th}$ interim as this determines the maximum recruitment possible for a stage $j,\;j=1,2,\ldots ,\;\max\left\{j_{\max},J-1\right\}$. Let us denote the maximum recruitment possible for a stage j as $\nu_{j}$. In other words, $\nu_{j}$ is the maximum recruitment that would be needed assuming all the current arms at stage j, continue until the end. Mathematically, if the number of arms present in stage j is $k^{*}$ (say), then the maximum recruitment possible for stage j is given by

$$\nu_{j}=\overset{J}{\underset{j}{\sum }}\overset{K}{\underset{k=0}{\sum }}n_{jk}\times \theta_{jk};\text{such that}\;\overset{K}{\underset{k=0}{\sum }}\theta_{jk}=k^{*}$$

Therefore, if all the treatment arms are present in stage j, then the maximum recruitment possible at stage j is $n_{\max}-n_{j-1}$. Intuitively it can be seen that, if the number of treatment arms present in any two stages (say j and $j^{\prime }$) remains the same, then the maximum total recruitment for the two stages will also be the same that is, $n_{j-1}+\nu_{j}=n_{j^{\prime }-1}+\nu_{j^{\prime }}$. In general, it can be said that the total maximum recruitment $\left(n_{j-1}+\nu_{j}\right)$ is a non‐increasing function of j.

#### 2.3.1 Uniform Recruitment

We consider a uniform recruitment pattern [8] with rate of recruitment λ. Usually, for this recruitment pattern, we obtain $t_{j}$ as $t_{j}=\frac{n_{j}}{\lambda }=\frac{t_{\max}}{n_{\max}}n_{j};\forall \;j$. We can then obtain the value of ${\tilde{t}}_{j}$ from Equation (1).

For this recruitment pattern, the expected number of pipeline participants at each interim analysis can be computed as,

$$\begin{array}{l} {ñ}_{j}=\left\{\begin{array}{ll} \lambda m_{0},\; & \text{if}\;{\tilde{t}}_{j}=t_{j}+m_{0}\\ \lambda m_{0},\; & \text{if}\;{\tilde{t}}_{j}={\tilde{t}}_{j-1}+m_{0}\\ \lambda ({\tilde{t}}_{j}-{\tilde{t}}_{j-1}),\; & \text{if}\;{\tilde{t}}_{j}=t_{\max} \end{array}\right. \end{array}$$

However, we must account for the fact that the number of pipeline participants recruited at each stage cannot surpass the maximum possible recruitment at stage j. Thus, for a given (ω,ψ), at analysis j, the number of pipelines can be determined as

$$\begin{array}{l} {ñ}_{j}=\left\{\begin{array}{ll} {ñ}_{j},\; & n_{j}\leq \nu_{j}\\ \nu_{j},\; & n_{j}>\nu_{j} \end{array}\right. \end{array}$$

This condition also ensures the total recruitment in the trial does not surpass the maximum sample size $n_{\max}$ as $\nu_{j}\leq n_{\max};\;\forall j$.

#### 2.3.2 Linear Recruitment

There can be trials for which uniform participant recruitment may poorly reflect recruitment patterns observed in practice. In multi‐centre trials, sites typically open gradually until a maximum number is reached. Thus recruitment rate may increase steadily and reach a uniform maximum. This has been referred to as a ‘Mixed Recruitment’ in previous work [9]. For this study, we consider a special case of the mixed recruitment: linear recruitment, where the recruitment rate never plateaus. This is considered as the extreme case, with mixed recruitment patterns leading to results in between the uniform and linear cases.

We assume that the recruitment rate is a linear function of time, say λ=Δt, where Δ is an unknown constant and $t=1,2,\ldots ,t_{\max}$. Then, in $t_{\max}$ units of time the number of recruitments, assuming this trend, is

$$\Delta \left(1+2+\cdots +t\right)=\frac{\Delta t_{\max}\left(t_{\max}+1\right)}{2}$$

 Equating this to $n_{\max}$ gives an estimate for Δ,

(3) $$\Delta =\frac{2n_{\max}}{t_{\max}\left(t_{\max}+1\right)}$$

Since, it takes $t_{j}$ units of time to recruit all $n_{j}$ patients at stage j, then, similarly, we have

$$\begin{array}{ll} & \;\Delta \frac{t_{j}\left(t_{j}+1\right)}{2}\;=n_{j}\\ & \Rightarrow t_{j}^{2}+t_{j}-\frac{2n_{j}}{\Delta }=0 \end{array}$$

Solving the quadratic for $t_{j}$ (restricting to the positive root since time is positive), we get

(4) $$t_{j}=-\frac{1}{2}+\frac{1}{2}\sqrt{1+\frac{8n_{j}}{\Delta }}$$

If ${\tilde{t}}_{j-1}\leq t_{j}$, the number of patients recruited after time $t_{j}$, during the $m_{0}$ units of time awaiting the outcome results is

$$\begin{array}{ll} {ñ}_{j} & =\Delta [(t_{j}+1)+(t_{j}+2)+\cdots +{\tilde{t}}_{j}]\\ & =\Delta m_{0}t_{j}+\Delta \frac{m_{0}\left(m_{0}+1\right)}{2} \end{array}$$

where values for Δ and $t_{j}$ can be acquired from Equations (3) and (4).

However, if ${\tilde{t}}_{j-1}>t_{j}$, then to compute the number of pipeline participants we replace $t_{j}$ in the above equation by ${\tilde{t}}_{j-1}$, that is,

$${ñ}_{j}\;=\Delta [\left({\tilde{t}}_{j-1}+1\right)+\left({\tilde{t}}_{j-1}+2\right)+\cdots +{\tilde{t}}_{j}]$$

Note that, if ${\tilde{t}}_{j-1}=t_{\max}$ then it is likely that the maximum number of patients have already been recruited in the trial, therefore, there will be no pipeline participants for the $j^{th}$ interim analysis and beyond.

Here again, the number of pipelines is constrained by the maximum possible recruitment and for a given (ω,ψ), at analysis j can be determined as

$$\begin{array}{l} {ñ}_{j}=\left\{\begin{array}{ll} {ñ}_{j},\; & n_{j}\leq \nu_{j}\\ \nu_{j},\; & n_{j}>\nu_{j} \end{array}\right. \end{array}$$

Similarly, this ensures the total recruitment in the trial is restricted by $n_{\max}$.

### 2.4 Final Sample Size

In order to arrive at the final sample size, consider the two following cases depending on the pipelines accrued due to the delay.

- Case I: When the delay length is relatively small such that ${n_{j}+ñ}_{j}<n_{j+1}\;\forall \;j$
  In this case, the number of pipeline participants is lower than the additional sample size required for the next interim analysis. Therefore, for all the stages these pipeline participants contribute directly to the next stage sample size (assuming the trial is not terminated at that stage). It inflates the final sample size only if the trial is stopped early due to futility or efficacy for all the treatment arms.
  For a given ω, if $n_{j}+{ñ}_{j}<n_{j+1}\forall j$, then,
$$\begin{array}{l} n_{\text{delay}}\left(\omega \right)=\left\{\begin{array}{ll} n_{j_{\max}}+{ñ}_{j_{\max}};\; & j_{\max}<J\\ n_{j_{\max}};\; & j_{\max}=J \end{array}\right. \end{array}$$
- Case II: When the delay length is larger.
  In this case the number of pipelines may surpass the next stage sample size that is, $n_{j}+{ñ}_{j}\geq n_{j+1}$. Here, the pipeline participants will contribute to a particular stage say $j^{*},j^{*}=j+1,j+2,\ldots j_{\max}$. In general, for any j and $j^{*}$, if $n_{j^{*}}\leq n_{j}+{ñ}_{j}<n_{j^{*}+1};j^{*}=j+1,j+2,\ldots j_{\max}$, then ${ñ}_{j}$ contributes to the ${j^{*}}^{th}$ stage sample size. ${ñ}_{j}$ inflates the final sample size iff, for some $j,n_{j}+{ñ}_{j}\geq n_{j_{\max}}$. This can happen for a single value of j or for a large delay length several values of $j\leq j_{\max}$.
  In order to compute the final sample size $n_{\text{delay}}(\omega )$, where the total recruitment has exceeded the value of $n_{j_{\max}}$ once or more than once that is, $n_{j}+{ñ}_{j}\geq n_{j_{\max}}$ for several values of j, we consider the ${ñ}_{j}$, that results in the highest total recruitment of $\left(n_{j}+{ñ}_{j}\right)$. which causes the highest drift from $n_{j_{\max}}$.
  In this case, the final sample size is given as
$$\begin{array}{ll} & n_{\text{delay}}\left(\omega \right)=n_{j_{\max}}+\max\left\{{\;n}_{j}+{\tilde{n}}_{j}-n_{j_{\max}};\forall \;j=1,2,\ldots ,j_{\max}\right\} \end{array}$$

The above gives expressions for the number of pipeline participants at each interim analysis assuming different recruitment patterns as well as the final sample size. Note that, in practice, if the delay is sufficiently large or the recruitment rate is relatively high, it is likely that for a $j<j_{\max}$, the pipeline participants recruited in the trial is sufficient to conduct all or at least some of the remaining interim analysis. This is particularly true if treatment arms are terminated for futility or efficacy in the interim analyses.

For example, consider a 4‐stage MAMS with 3 experimental treatment arms. Here, we take the instance where ω takes the values (1,1,3) say. Thus, the number of treatment arms present in the 4 stages are 4, 2, 0 and 0 respectively. Now, let us assume it takes 50 patients in each arm at each stage to conduct the interim stages for the specific power requirement of the trial, that is, $n_{jk}=50\;\forall \;j,k$. Hence, the maximum sample size for the trial is $n_{\max}=800$ and at each interim a maximum of 200 patients is required. Assume, it takes 2 years $\left(t_{\max}=24\right)$ to recruit all 800 patients. Then assuming a uniform recruitment, the recruitment rate is given as approx. λ=33 patients per month. Now, if $m_{0}=12$, that is, it takes 1 year to observe the treatment outcome, then, at the first interim analysis while we await the patient outcome for the 200 first stage patients, the number of pipeline participants recruited in the trial is 400 $\left({ñ}_{1}\right)$. Here, at the first interim, two treatment arms are terminated. Hence, for the remaining 3 stages, the maximum sample size required based on the remaining single treatment arm along with the control would be 300. In this case, the recruited sample size by the time of the first interim surpasses the maximum required sample size for stage 2 and above. This implies that, it is most likely that the recruitment would be stopped at the end of the first interim analysis, where the trial has already recruited a total of 600 patients. This would be sufficient to conduct the remaining interim analyses. Hence, the number of pipeline participants ${ñ}_{j},\;j=2,3$ becomes 0. Thus, in this case, given ω=(1,1,3), the final sample size $n_{\text{delay}}\left(\omega \right)=600$, instead of n(ω)=400. Therefore, the expression given in the above section for $n_{\text{delay}}\left(\omega \right)$ would result in the same value of $n_{\text{delay}}\left(\omega \right)$ even if we consider ${ñ}_{j}=0\;\text{for}\;j=2,3.$ The codes to obtain the number of pipeline participants and the efficiency thus lost can be found at https://github.com/AritraMukherjee/Delay‐in‐MAMS.

### 2.5 Example Scenarios

In order to investigate the EL for different delay lengths we have considered the following simulation scenario. For our study, we have considered both equally and unequally spaced interim analyses under uniform and linear recruitment pattern. The results for the EL are obtained assuming a target interesting treatment effect of 0.5. The efficacy stopping boundaries are O'Brien and Fleming boundaries [14] and the futility boundaries are fixed at the value 0 (equivalent to stopping for futility if the one‐sided *p*‐value is ≥ 0.5). The FWER and (conjunctive) power values are maintained at 5% and 80%. We have assumed a total recruitment time of 36 months and obtained the EL for 0, 6, 12, 18, 24, 30, and 36 months of delay for different values of J and K. We have limited our simulations to a maximum of K=4 and J=4 [i.e., a 4 stage‐5 arm trial (4 experimental and 1 control arm)] due to computational complexities thereafter.

For unequally spaced MAMS, we have considered three different scenarios. First we consider an equally spaced design (Interim spacing I). Then a design for which the first interim is conducted before the first interim of an equally spaced design and the remaining interims are spaced equally thereafter (Interim spacing II). For example, for a 4‐stage design, the interim spacing II is given as $\left(\frac{1}{5},\frac{7}{15},\frac{11}{15},1\right)$ where, the first interim is conducted after results for 20% of the total sample size is observed instead of 25% of an equally spaced design. Thereafter, the interims are conducted at equally spaced interval. For example, the second interim will be conducted after we observe results for ${\frac{7}{15}}^{th}\left[=\frac{1}{5}+\left(1-\frac{1}{5}\right)*\frac{1}{3}\right]$ of the total sample size. Similarly, the third interim will be conducted after we observe results for ${\frac{11}{15}}^{th}\left[=\frac{1}{5}+\left(1-\frac{1}{5}\right)*\frac{2}{3}\right]$ of the total sample size. Lastly, we consider a design for which the first interim is conducted after the first interim of an equally spaced design and the remaining interims are spaced equally thereafter (Interim spacing III). The exact values of interim spacing options for different J's are given in Table 1.

**TABLE 1:** Interim spacing across different K‐arm trials for different values of J.

Interim spacing	J=2	J=3	J=4
I	$\left(\frac{1}{2},1\right)$	$\left(\frac{1}{3},\frac{2}{3},1\right)$	$\left(\frac{1}{4},\frac{2}{4},\frac{3}{4},1\right)$
II	$\left(\frac{7}{20},1\right)$	$\left(\frac{1}{4},\frac{5}{8},1\right)$	$\left(\frac{1}{5},\frac{7}{15},\frac{11}{15},1\right)$
III	$\left(\frac{3}{4},1\right)$	$\left(\frac{1}{2},\frac{3}{4},1\right)$	$\left(\frac{1}{2},\frac{4}{6},\frac{5}{6},1\right)$

## 3 Results

### 3.1 Equally Spaced Interim Analyses

**FIGURE 2:**
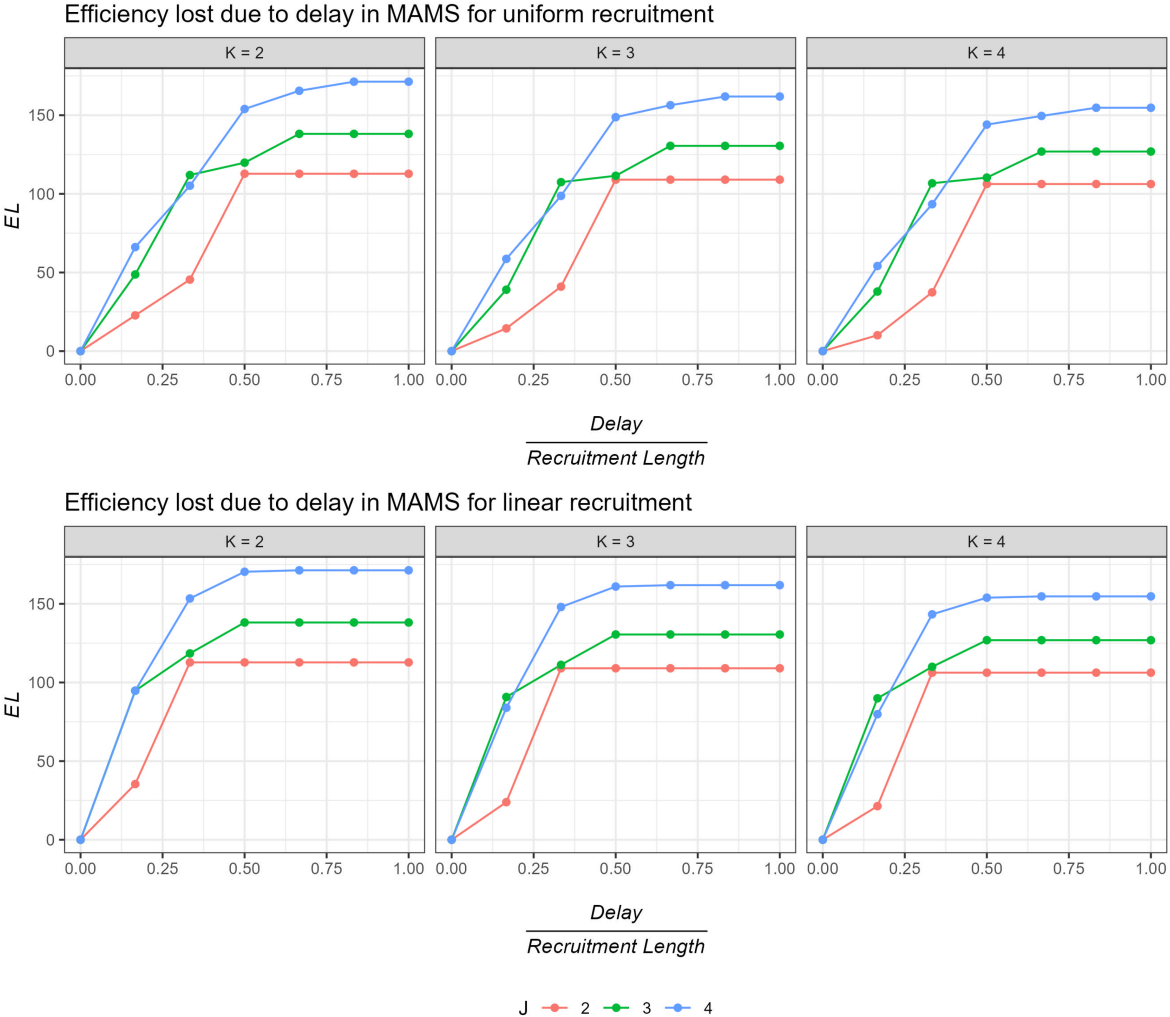
Efficiency loss (EL) for different stages across different number of treatment arms (K) for uniform and linear recruitment. Here, we assume the global alternative hypothesis is true, that is, all treatment arms are effective.

**FIGURE 3:**
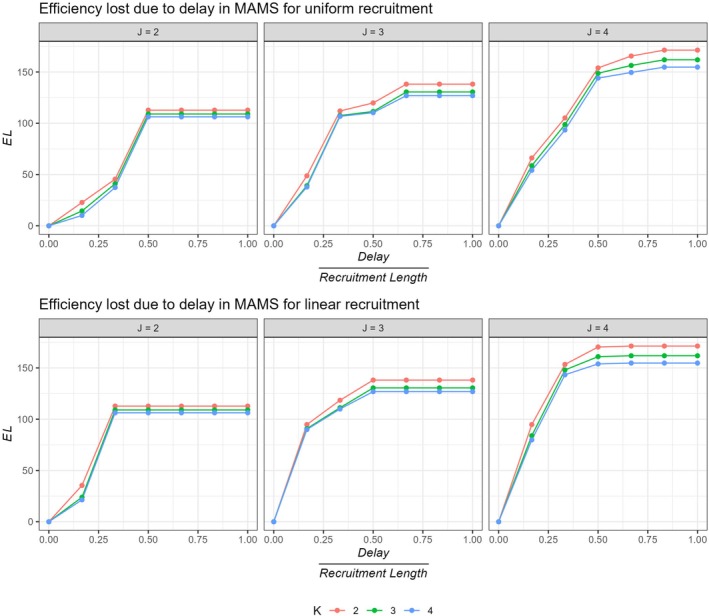
Efficiency loss (EL) for different stages across different number of stages (K) for uniform and linear recruitment. Here, we assume the global alternative hypothesis is true, that is, all treatment arms are effective.

Here, the EL values are plotted in Figures 2 and 3, computed under the global alternative, assuming all treatment arms are effective for both uniform and linear recruitment.

Figure 2 shows that the EL is quite dependent on the number of stages J. A 2‐stage design is likely to suffer the least loss of efficiency due to delay. The pipeline participants contributing to the trial increases with the number of stages. Especially for smaller delay lengths, a 2‐stage design shows significantly lower EL values. This can also be due to the fact that in general the efficiency gain from using a 2‐stage design is lower, thus the loss in efficiency does not scale as much as the same in a 3 or 4 stage trial. For lower delay lengths, the $ESS_{\text{delay}}$ for 3 or 4 stage designs for both uniform and linear recruitment is relatively lower compared to a 2‐stage design.

It can be observed from Figure 3, for same number of stages J, the EL seems to be similar across different number of arms for different delay lengths. This might be because of the proportion of pipeline participants being recruited, as compared to the maximum sample size, remains similar. The EL is almost identical for K=3 and 4 especially for lower delays, differing very marginally for higher delay lengths. A 5‐arm (4 treatment +control) trial seem to perform slightly better as compared to a 4‐arm (3 treatment +control) trial in terms of a marginally lower EL. For a given number of stages, usually a lower number of treatment arms undergo a relatively greater EL.

**FIGURE 4:**
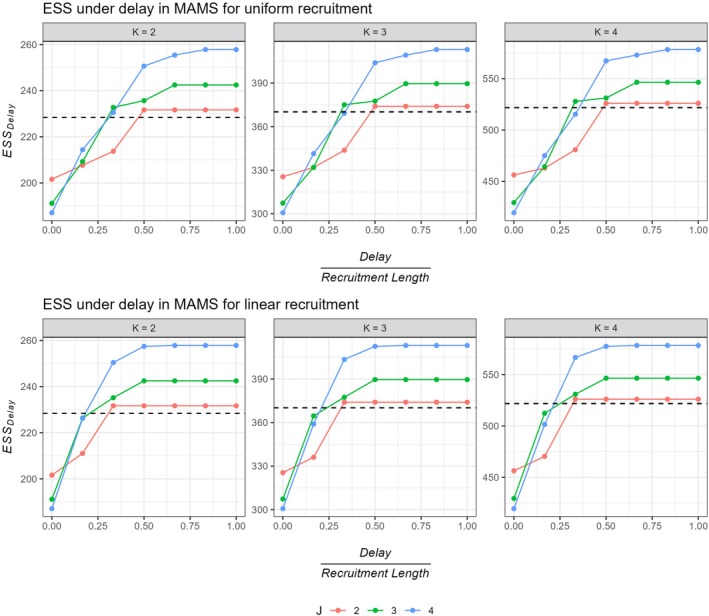
$ESS_{\text{delay}}$ for different stages across different number of stages (K) for uniform and linear recruitment. Here, we assume the global alternative hypothesis is true, that is, all treatment arms are effective. The black dashed line indicates the sample size of a single stage design under the same design setting.

Furthermore, Figure 4 plots the ESS values accounting for delay for MAMS with different number of stages across different values of K. This can be another way to look at the efficiency lost due to delay. The figure contrasts the ESS under delay with the sample size of a single stage design (black dashed line in the figure), which demonstrates efficiency losses prominently. It can be observed that for delay lengths greater than 33% of the total recruitment length for a uniform recruitment, MAMS recruits more participants than a single stage design on average, especially for K>2. This proportion further reduces to 16.66% for a linear recruitment rate.

### 3.2 Unequally Spaced Interim Analyses

To observe the effect of delayed outcome on the interim spacing, we also obtained EL assuming different delay lengths and different interim spacings.

**FIGURE 5:**
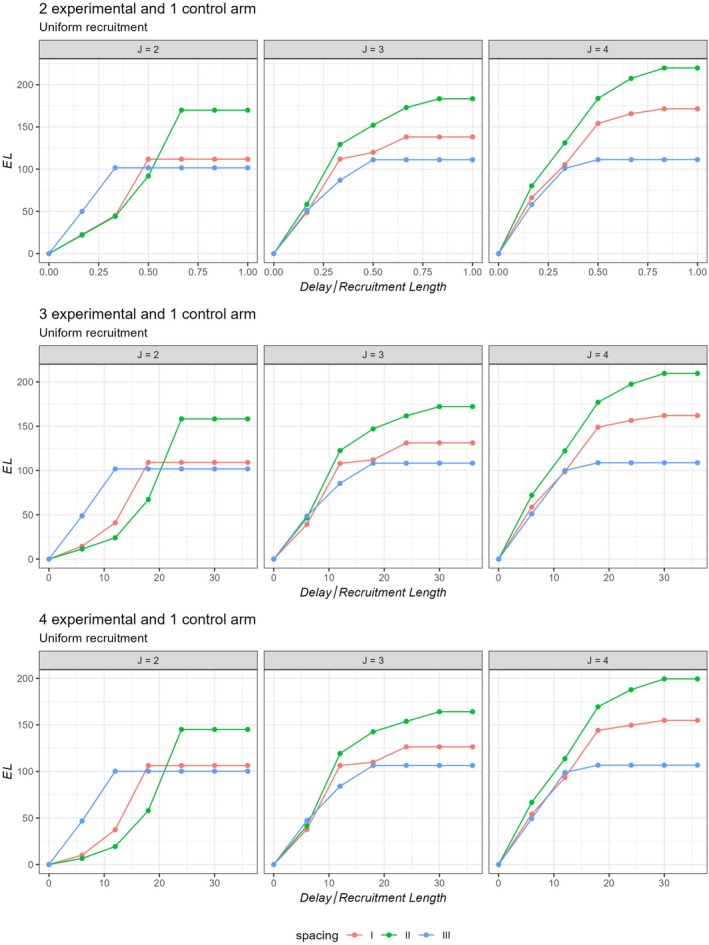
Efficiency loss (EL) for unequally spaced MAMS for different values of K and J assuming Uniform recruitment.

**FIGURE 6:**
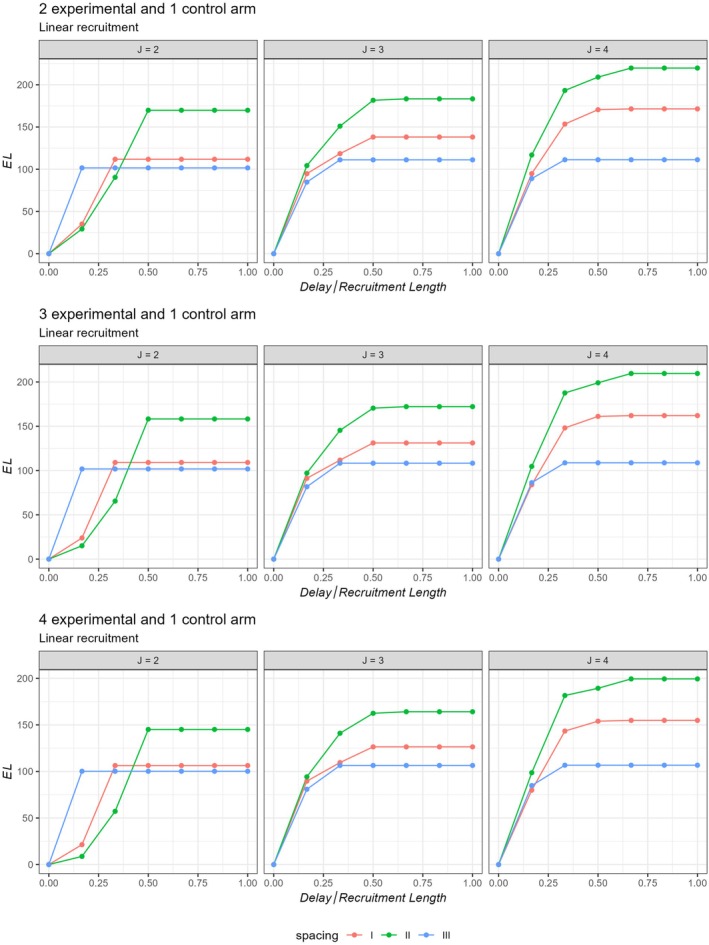
Efficiency loss (EL) for unequally spaced MAMS for different values of K and J assuming Linear recruitment.

Figures 5 and 6 plot the EL values for unequally spaced MAMS for different delay lengths for uniform and linear recruitment patterns respectively. It can be observed from these figures that EL across different numbers of arms (K) remains similar. The figures highlight that the loss in efficiency is the minimum when the interims are pushed to the latter end of the trial, especially for large delay lengths $\left[m_{0}\geq t_{\max}/3\right]$. This is likely due to the fact that, when the interims are conducted at a later stage of the trial, most of the patients have already been recruited to the trial. This leaves a lower number of pipeline participants to be recruited in the trial. Therefore, the inflation in the $ESS_{\text{delay}}$ as compared to ESS is limited, resulting in a relatively lower EL. The exact EL values are provided in Supporting Information. Note that, when EL is plotted for different delay lengths assuming symmetric stopping boundaries, the findings indicate that pushing the first and subsequent interims to the latter end of the design can be harmful for the efficiency of the trial. See Supporting Information for these findings.

## 4 Example

We consider the TAILoR trial [EudraCT no.: 2012‐000935‐18] to illustrate the impact a delayed outcome may have on the design efficiency. TAILoR was a dose‐ranging trial, that aimed to assess whether telmisartan can reduce insulin resistance in HIV‐positive individuals being treated with combination antiretroviral therapy (cART, [15]). The trial was conducted in 19 UK Sexual Health Clinics and/or HIV treatment centres from February 2013 until July 2015 (Recruitment start date: 21st February 2013). The target recruitment figure of 370 was computed based on a required sample size of 336 accounting for 10% drop‐out rate. The primary outcome was the reduction in insulin resistance measured by HOMA‐IR scale after 24 weeks from baseline, that is, $m_{0}=6$ The trial conducted an interim analysis when the primary data was available for 168 patients. The trial recruited a total of 379 patients who were randomised across the treatment arms with (1:1:1:1) allocation ratio. The design, if not stopped in stage 1, would have required a minimum of 84 samples to conduct stage 2 (42 patients in the control and one experimental treatment arm). As per the design, recruitment continued whilst the treatment outcome was awaited. Assuming a uniform recruitment, this would result in the number of pipeline participants being approximately 78. In this case, the EL is found to be 16% under a uniform recruitment and 35% for a linear one. However, if the recruitment rate was a little faster than what was observed, it could have meant considerable efficiency losses for the design. Consider the following scenario under the same design considerations to observe the EL. Let us assume it takes 24 months to recruit all 379 patients instead of 29 months. Here, $t_{\max}=24$ weeks instead of 29. This implies a recruitment rate of 16 patients per month approximately, recruiting 95 patients as pipeline participants under Uniform recruitment. This number further inflates to a maximum of 149, under a linear recruitment assumption. Therefore, the EL observed in this case becomes 23% under uniform recruitment and 58% for linearly increasing recruitment rate. For a lower value of $t_{\max}$, this value of EL quickly rises. This can be observed from Table 2.

**TABLE 2:** EL values for different recruitment rates and recruitment patterns for the TAILoR trial.

Stopping boundary	$t_{\max}$	$m_{0}$	No. of arms present in stage 2	$n_{\text{delay}}(\omega )$ (uniform)	$n_{\text{delay}}(\omega )$ (linear)	ESS	$ESS_{\text{delay}}$ (uniform)	$ESS_{\text{delay}}$ (linear)	$EL_{Uniform}$	$EL_{Linear}$
Original	29	6	0	69.52	112.15	279.69	290.69	303.45	14.67	31.64
			2	0.00	28.15					
	24	6	0	84.00	138.97	279.69	292.99	317.05	17.71	49.71
			2	0.00	54.97					
			3	0.00	12.97					
	20	6	0	100.80	168.00	279.69	299.23	336.00	25.91	74.61
			2	16.80	84.00					
			3	0.00	42.00					
OBF	29	6	0	73.77	119.02	323.15	327.66	334.23	14.17	34.78
			2	0.00	29.87					
	24	6	0	89.16	147.51	323.15	328.68	343.12	17.08	62.28
			2	0.00	58.35					
			3	0.00	13.76					
	20	6	0	107.06	178.44	323.15	332.06	356.68	27.53	104.44
			2	17.84	89.22					
			3	0.00	44.61					
Pocock	29	6	0	80.23	129.44	292.74	316.73	339.98	38.44	75.71
			2	0.00	32.49					
	24	6	0	96.96	160.41	292.74	321.77	361.03	46.67	109.86
			2	0.00	63.45					
			3	0.00	14.97					
	20	6	0	116.36	193.93	292.74	332.60	387.87	64.43	153.76
			2	19.39	96.96					
			3	0.00	48.48					
Triangular	29	6	0	78.07	125.96	293.64	313.34	333.40	32.06	64.69
			2	0.00	31.62					
	24	6	0	94.29	155.98	293.64	317.39	352.29	38.70	95.44
			2	0.00	61.70					
			3	0.00	14.55					
	20	6	0	113.11	188.52	293.64	326.74	376.91	53.89	135.15
			2	18.85	94.26					
			3	0.00	47.13					

*Note:* The unit of time is months in the following table. The ESS given is derived assuming a true global alternative (all treatments are effective). This provides the maximum EL for different stopping boundaries.

We repeated the process for different types of stopping boundaries to observe the possible EL values. Different scenarios (e.g., true global null, LFC) have also been considered for evaluating the loss. These results are presented in Supporting Information.

## 5 Discussion

Adaptive trial designs have been proven to be efficient trial design options when compared to traditional parallel arm RCTs. MAMS designs in particular have been gaining momentum in its usage due to the efficiency they provide in terms of a reduced ESS as compared to conducting multiple two‐arm clinical trials. While previous work discusses the adverse effect a delayed outcome has on adaptive designs, little work has been done in the context of a MAMS design. In this study we therefore seek to quantify the loss experienced by MAMS trials due to delayed outcomes. To our knowledge, there has been no study that formally provides a methodology to compute the number of pipeline participants recruited in a trial under the MAMS setting. We have developed this methodology to estimate the number of pipeline participants for a MAMS design assuming different recruitment patterns. We quantify the inflation in the ESS as a result of accumulated pipeline participants through a metric EL. We consider results assuming both equally and unequally spaced interim analyses.

Results indicate that a delayed outcome has the potential to reduce the expected efficiency gain of a MAMS design significantly. As observed in the case of GSD, here also, we see heavy losses for MAMS as delay length increases. For a small $m_{0}(<18\text{months})$ as compared to the total maximum recruitment length, that is, for $m_{0}$ less than 50% of the maximum recruitment length, a 2‐stage design incurs minimum loss. The plots further revealed that, trials with uniform recruitment tend to have lower EL values as compared to a trial with linear recruitment. This is directly linked to the number of pipeline participants, which are on average greater for a linearly increasing recruitment trend. We observe that, a trial might end up recruiting its maximum sample size $\left(n_{\max}\right)$, as $m_{0}$ comes closer to $\frac{t_{\max}}{2}$ for uniform patient accrual. For a linear recruitment, this happens sooner, at 1/3rd of the total recruitment period $t_{\max}$. In this case, we observe maximum EL, crossing the mark of 100%, that is, all of the expected EG is lost due to a delayed outcome. A relatively low value of EL is observed when $m_{0}\leq 6$ for K=3 and 4 and for $m_{0}\leq 12$ for K=2. It can be said that, a MAMS design provides significant benefit compared to a multi arm single stage trial if the delay length is less than 20% of the total recruitment length for more than 3 treatment arms and 33% of the total recruitment for a 3‐arm multistage trial. For a linear recruitment pattern, the threshold for delay becomes more strict (less than 16.6% of the total recruitment length).

We also have disentangled the relationship between a long‐term primary treatment outcome and the spacing of the interims. Three different interim combinations were considered for every J, where, either the interims were equally spaced, or, the first interim was done sooner than an equally spaced design, or the first interim was done later than that of an equally spaced design. The findings reconfirm the previous research of Mukherjee et al. [9]. The maximum EL was observed when the first interim is conducted earlier than an equally spaced design. The primary reason for this is the choice of stopping boundaries being fixed at 0. This resulted in the following two: 1. We observed a higher ESS for trials with spacing option II in Table 1 as opposed to option I. The high ESS in turn resulted in a considerably low EG without delay, reducing the denominator in EL. 2. The selected stopping boundaries generated a design for interim spacing II in Table 1 with a much higher maximum sample size as compared to an equally spaced design. This high maximum sample size resulted in a possibility of recruiting more pipeline participants that increased the difference in the EG with and without delay (i.e., increased the numerator of EL). Due to the combination of 1 and 2, that inflation in the EL value for interim spacing option II increased significantly. Therefore, conducting the interims earlier than an equally spaced design, resulted in higher EL as compared to an equally spaced design as well as for the designs when interims are conducted at a later stage. The only scenario, where conducting the interims earlier were found to be useful was for 2‐staged designs with relatively lower delay lengths $\left[m_{0}\leq t_{\max}/2\right]$. Therefore, conducting the first interim later might be a better choice in presence of a delayed outcome. While the EL is lower as compared to an equally spaced design, care should be taken to choose an appropriate interim spacing as $ESS_{\text{delay}}$ for interim spacing III might still be higher than interim spacing I.

Further evaluations reveal that if the number of effective treatment arms increases, the EL also increases [See Supporting Information for the figures]. This likely is due to the fact that, as more treatment arms become effective, the probability of stopping the trial early for efficacy increases. In this case there is a greater chance that pipelines will be accumulated in the treatment arms in presence of delayed outcome and contribute to the EL with a higher probability. Therefore, the EL observed assuming the global alternative is true gives us the maximum EL among all possible cases.

We have considered uniform recruitment to model single‐centred small scale clinical trials. However, this might poorly reflect reality, especially for multi‐centred large‐scale trials. Here, a mixed recruitment is more suitable to model the recruitment pattern [9]. In this article, we have focused on linear recruitment only, as an extreme for the aforementioned recruitment pattern. This likely gives us a maximum value for EL for conducting MAMS in presence of delay.

While the present work sheds light only on continuous outcomes, the methodology can be adapted easily for other types of non‐normal treatment outcomes with fixed delay lengths. However, the threshold for obtaining significant benefit of using a MAMS design might be different for different endpoints. Since, the method to obtain pipeline participants does not differ across different types of endpoints, the value of $ESS_{\text{delay}}$ can be easily computed given the stopping probabilities for different sets of possible trial outcomes. However, if the delay length or the time to observe the primary outcome is variable (e.g., in the time‐to event outcome case) computing pipeline participants might not be straightforward. This remains an area for future research.

In some settings (e.g., in the pharmaceutical industry), the expected sample size may be less of interest compared to the expected time to make a decision. In our previous study with group sequential design [9] with fixed delay, it was found that if the metric of assessing the efficiency of a design is the expected time to complete the trial, a group sequential design on average always outperforms a single stage design. Since here, the design we have considered is a group‐sequential MAMS, the result readily extends to this design setting.

There have been recent developments in MAMS trials where one adds additional treatments in an ongoing trial known as platform trials [16, 17, 18, 19]. A long‐term primary outcome can be harmful for the adaptations in such trials similarly to MAMS. While the method to obtain the number of pipeline participants can be adapted from this study, our conclusions might not be applicable directly. This is primarily because the metric to assess the efficiency of a platform trial is not always the expected sample size. Therefore, we still need to explore the underlying effect a delayed outcome might have on this class of MAMS trials, an active area of future research for us.

In summary, it can be concluded that a long‐term primary outcome can be harmful to the efficiency of a MAMS design if recruitment is continued while treatment outcome is awaited. Most of the expected efficiency gain is lost due to delay if the delay length is relatively large compared to the total recruitment length. The results indicate little to no benefit in utilising a MAMS design if the delay length is more than 33% of the total recruitment length under a uniform recruitment pattern, particularly for designs with more than two stages. This threshold further reduces significantly under a more rapid recruitment pattern like linear recruitment. To retain a sizable amount of the efficiency gain, the delay preferably should be less than 25% of the total recruitment length. Some of this lost efficiency can be saved if the first interim analysis is conducted later than the first interim of an equally spaced design. However, caution should be taken in selecting appropriate design spacings as this can still result in a higher ESS.

## Conflicts of Interest

The authors declare no conflicts of interest.

## Supporting information


**Data S1.** Supporting Information.

## Data Availability

The data that support the findings of this study are openly available in Github at https://github.com/AritraMukherjee/Delay‐in‐MAMS.
