# Supplementary material for: Impact of Endpoint Delay on the Efficiency of Multi Arm Multi Stage Trials
Source: Stat Med. 2025 Sep 15;44(20-22):e70245. doi: 10.1002/sim.70245 (PMC12436084; doi:10.1002/sim.70245)
Supplement: Supplementary file 1 — Data S1. Supporting Information. [file SIM-44-0-s001.pdf]

Received: Added at production | Revised: Added at production | Accepted: Added at production

DOI: xxx/xxxx

ARTICLE TYPE

Supplementary materials for “Impact of endpoint delay on the efficiency of Multi Arm Multi Stage trials"

1 | METHODS: EVALUATING THE PROBABILITY $[P(\omega, \psi|\tau, E, F)]$ FOR A PARTICULAR OUTCOME IN MAMS DESIGN

Z_{jk} denotes the test statistic for k^{th} treatment in stage j and $\tau = \left(\tau_1, \tau_2, \dots, \tau_K\right)$, denote the vector of the true treatment effects. Then, the mean of Z_{jk} and covariance between Z_{jk} and $Z_{j'k'}$ are given as $E(Z_{jk})$ and $cov(Z_{jk}, Z_{j'k'})$ [see section 2.1 of the paper for the exact expression of Z_{jk}, $E(Z_{jk})$ and $cov(Z_{jk}, Z_{j'k'})$]

Let $Z_j = (Z_{j1}, Z_{j2}, \dots, Z_{jK})'$ denote the vector of all test statistics for K treatment arms at stage j and $Z = (Z_1, Z_2, \dots, Z_J)'$ denote the vector of all the test statistic (for all $j \in 1, \dots, J$. The mean and variance of the aforementioned vectors can be represented as $E(Z)$ and $cov(Z, Z)$. Further, the elements of the mean vector and covariance matrix can be easily computed from the expressions of $E(Z_{jk})$ and $cov(Z_{jk}, Z_{j'k'})$, provided in the main paper.

Let, $P(\omega, \psi|\tau, e, f)$, denote the probability of a particular value of $(\omega, \psi)\epsilon(\Omega, \Psi)$ given the stopping boundaries (e, f) and treatment effect τ.

Then, $P(\omega, \psi|\tau, e, f)$ can be computed from the JK-dimensional integral given as

$$P(\omega, \psi|\tau, e, f) = \int\limits_{l(1,1,\psi,\omega,e,f)}^{u(1,1,\psi,\omega,e,f)} \cdots \int\limits_{l(K,1,\psi,\omega,e,f)}^{u(K,1,\psi,\omega,e,f)} \cdots \int\limits_{l(1,J,\psi,\omega,e,f)}^{u(1,J,\psi,\omega,e,f)} \cdots \int\limits_{l(K,J,\psi,\omega,e,f)}^{u(K,J,\psi,\omega,e,f)} \phi\{x, E(Z), cov(Z, Z)\} dx_{KJ} \dots dx_{1J} \dots dx_{K1} \dots dx_{11}.$$

where, $\phi\{x, \mu, \Sigma\}$ is the pdf of a multivariate normal distribution with mean μ, variance Σ evaluated at the vector $x = (x_{11}, \cdots, x_{KJ})'$.

Here, the boundaries of the integral are determined as

$$l(k, j, \psi, \omega, e, f) = \begin{cases} e_j & : \psi_k = 1, \omega_k = j, \\ -\infty & : \{\omega_k < j\} \cup \{\psi_k = 0, \omega_k = j\}, \\ f_j & : otherwise. \end{cases}$$

$$u(k, j, \psi, \omega, e, f) = \begin{cases} f_j & : \psi_k = 0, \omega_k = j, \\ \infty & : \{\omega_k < j\} \cup \{\psi_k = 1, \omega_k = j\}, \\ e_j & : otherwise \end{cases}$$

The value can be evaluated to find the probability of a particular outcome of the MAMS design, which thereafter contributes to evaluate the expected sample size.

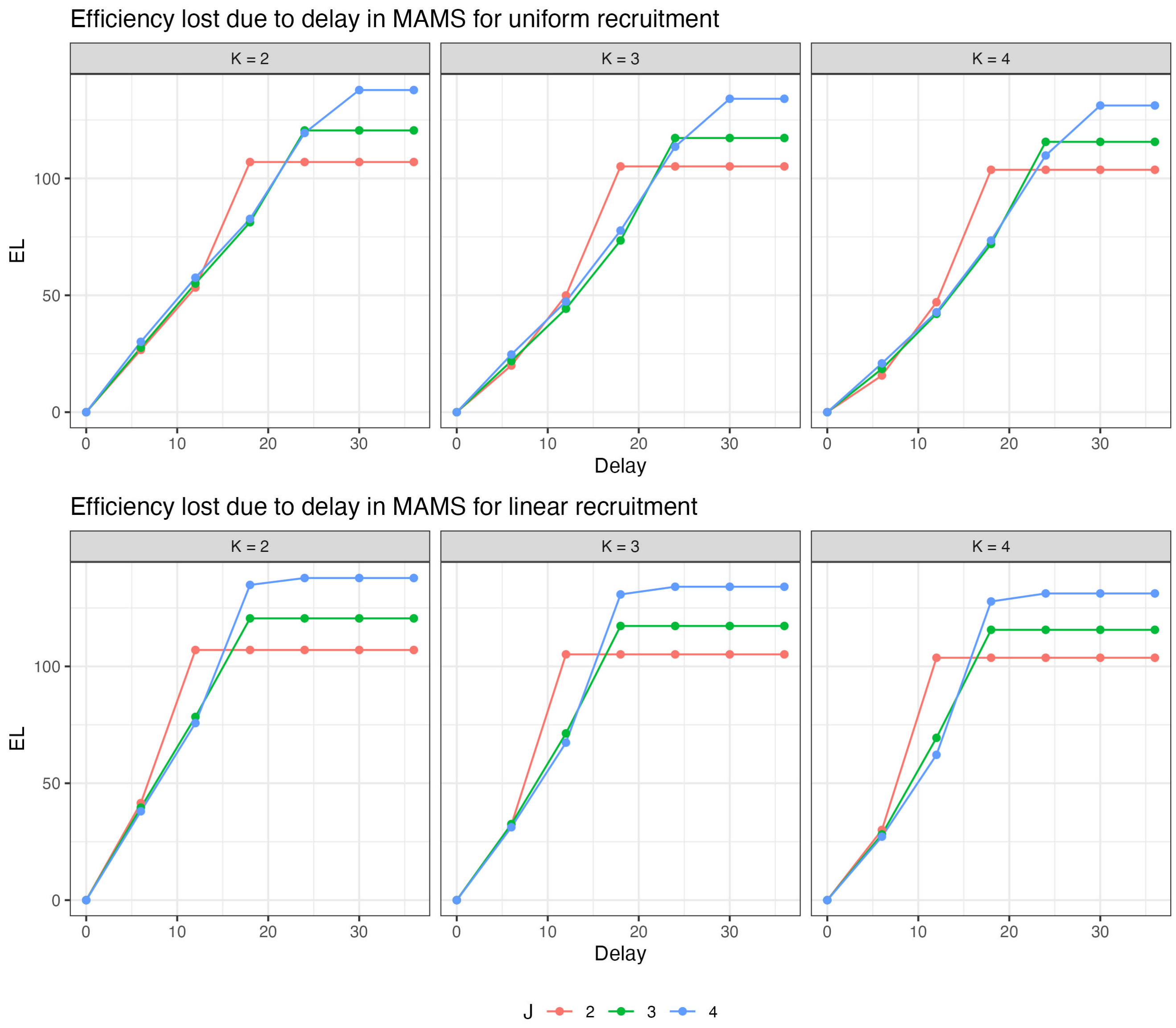

Figure 1 Efficiency loss (EL) for different stages across different number of treatment arms (K) for uniform and linear recruitment. Here, we assume the global null hypothesis is true, i.e. all treatment arms are ineffective.

2 | RESULTS UNDER THE GLOBAL NULL AND LEAST FAVOURABLE CONFIGURATION

Figures 1 and 2 describes the EL values for other alternative situations where we assume a true global null and the LFC. Under LFC, without loss of generality, we assume that treatment 1 is effective and the remaining treatment arms are not.

For figure 1, it can be seen that the EL is the minimum among the true global alternative and least favourable configuration. The expected efficiency gain is relatively low in this case and we observe a much less EL.

Figure 2 plots the same for the LFC. It can be observed that, in this case, the value of EL decreases further as compared to a true global alternative. In this case, there is a lower chance of terminating the trial early due to a higher number of ineffective treatment arms. Therefore, the number of pipelines contributing to the inflation in ESS reduces.

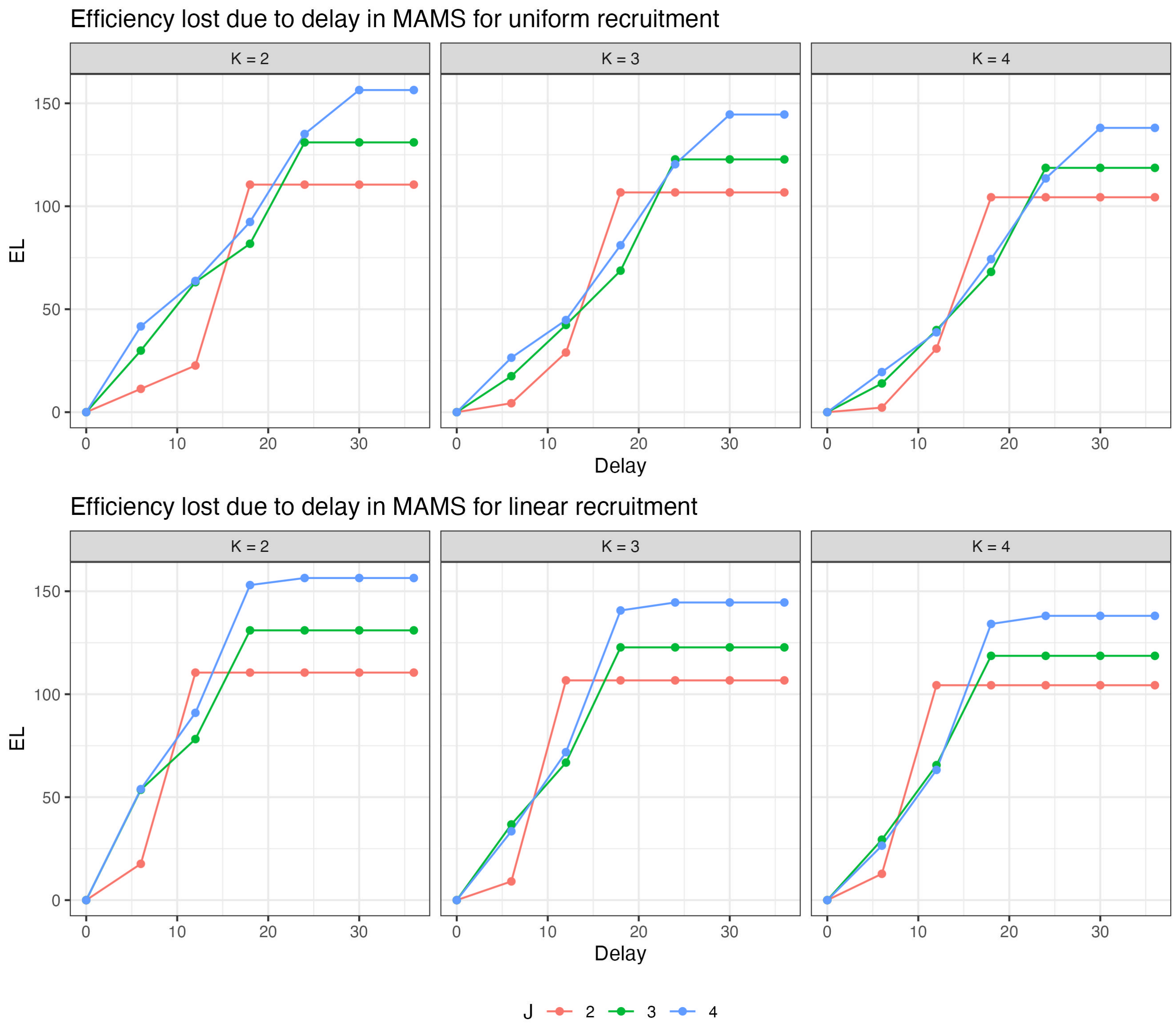

Figure 2 Efficiency loss (EL) for different stages across different number of treatment arms (K) for uniform and linear recruitment. Here, we assume only one treatment arm is effective (WLOG, we consider treatment 1 to be effective).

3 | EXACT EL VALUES FOR MAMS DESIGNS UNDER THE TRUE GLOBAL ALTERNATIVE HYPOTHESIS

3.1 | Equally spaced interim analyses

The following section provides exact values of EL under the true alternative hypothesis assumption.

Table 1 Efficiency loss (EL) values for different delay lengths assuming uniform and linear recruitment. Here, the sample sizes are obtained assuming conjunctive power restrictions and a true global alternative,i.e. all treatments are effective.

K	n_{single}	J	n_{max}	ESS	Delay	ESS_{delay} (Uniform)	ESS_{delay} (Linear)	$EL_{Uniform}$	EL_{Linear}
2	228.39	2	231.68	201.60	0	201.60	201.60	0.00	0.00
					6	207.66	211.04	22.62	35.23
					12	213.72	231.67	45.25	112.24
					18	231.67	231.67	112.24	112.24
					24	231.67	231.67	112.24	112.24
					30	231.67	231.67	112.24	112.24
					36	231.67	231.67	112.24	112.24
		3	242.49	191.17	0	191.17	191.17	0.00	0.00
					6	209.27	226.36	48.62	94.55
					12	232.75	235.17	111.73	118.22
					18	235.67	242.48	119.55	137.85
					24	242.48	242.48	137.85	137.85
					30	242.48	242.48	137.85	137.85
					36	242.48	242.48	137.85	137.85
		4	257.83	187.08	0	187.08	187.08	0.00	0.00
					6	214.39	226.21	66.10	94.74
					12	230.51	250.42	105.14	153.34
					18	250.65	257.44	153.90	170.33
					24	255.43	257.82	165.47	171.26
					30	257.82	257.82	171.26	171.26
					36	257.82	257.82	171.26	171.26
3	370.20	2	374.03	325.49	0	325.49	325.49	0.00	0.00
					6	331.92	336.12	14.38	23.78
					12	343.73	373.99	40.80	108.47
					18	373.99	373.99	108.47	108.47
					24	373.99	373.99	108.47	108.47
					30	373.99	373.99	108.47	108.47
					36	373.99	373.99	108.47	108.47
		3	389.44	307.37	0	307.37	307.37	0.00	0.00
					6	331.98	364.54	39.24	91.17
					12	375.04	377.41	107.91	111.68
					18	377.59	389.57	111.98	131.07
					24	389.57	389.57	131.07	131.07
					30	389.57	389.57	131.07	131.07
					36	389.57	389.57	131.07	131.07
		4	412.99	300.73	0	300.73	300.73	0.00	0.00
					6	341.44	358.97	58.59	83.84
					12	369.24	403.43	98.62	147.83
					18	403.95	412.46	148.58	160.82
					24	409.25	413.07	156.20	161.71
					30	413.07	413.07	161.71	161.71
					36	413.07	413.07	161.71	161.71
4	521.74	2	526.08	456.33	0	456.33	456.33	0.00	0.00

…Table 1 continued from previous page

			6	462.96	470.36	10.14	21.46
			12	480.86	526.05	37.50	106.59
			18	526.05	526.05	106.59	106.59
			24	526.05	526.05	106.59	106.59
			30	526.05	526.05	106.59	106.59
			36	526.05	526.05	106.59	106.59
3	546.47	429.40	0	429.40	429.40	0.00	0.00
			6	464.36	512.39	37.80	89.72
			12	527.88	530.86	106.48	109.70
			18	531.20	546.48	110.07	126.59
			24	546.48	546.48	126.59	126.59
			30	546.48	546.48	126.59	126.59
			36	546.48	546.48	126.59	126.59
4	578.21	419.54	0	419.54	419.54	0.00	0.00
			6	475.08	501.46	54.36	80.19
			12	515.40	566.62	93.83	143.97
			18	567.34	577.48	144.68	154.60
			24	572.99	578.34	150.20	155.44
			30	578.34	578.34	155.44	155.44
			36	578.34	578.34	155.44	155.44

3.2 | Unequally spaced interim analyses

Table 2 Efficiency loss (EL) values for different delay lengths, for a 3-arm MAMS with different values of J and unequally spaced interims, assuming uniform and linear recruitment. Here, the sample sizes are obtained assuming conjunctive power restrictions and a true global alternative,i.e. all treatments are effective. Here I, II and III represents the first row of table 1. The EL values are computed based on the equivalent single stage sample size of 228.39.

J	Spacing	n_{max}	ESS	Delay	ESS_{delay} (Uniform)	ESS_{delay} (Linear)	$EL_{Uniform}$	EL_{Linear}
2	I	231.57	201.53	0	201.53	201.53	0.00	0.00
				6	207.58	210.96	22.54	35.10
				12	213.64	231.57	45.08	111.84
				18	231.57	231.57	111.84	111.84
				24	231.57	231.57	111.84	111.84
				30	231.57	231.57	111.84	111.84
				36	231.57	231.57	111.84	111.84
	II	240.11	211.61	0	211.61	211.61	0.00	0.00
				6	215.30	216.52	22.00	29.26
				12	218.99	226.78	44.01	90.44
				18	227.02	240.11	91.86	169.84
				24	240.11	240.11	169.84	169.84
				30	240.11	240.11	169.84	169.84
				36	240.11	240.11	169.84	169.84
	III	228.78	204.20	0	204.20	204.20	0.00	0.00
				6	216.28	228.78	49.92	101.61
				12	228.78	228.78	101.61	101.61

…Table 2 continued from previous page

				18	228.78	228.78	101.61	101.61
				24	228.78	228.78	101.61	101.61
				30	228.78	228.78	101.61	101.61
				36	228.78	228.78	101.61	101.61
3	I	242.57	191.23	0	191.23	191.23	0.00	0.00
				6	209.34	226.44	48.74	94.77
				12	232.84	235.26	111.98	118.49
				18	235.75	242.57	119.82	138.15
				24	242.57	242.57	138.15	138.15
				30	242.57	242.57	138.15	138.15
				36	242.57	242.57	138.15	138.15
	II	256.77	194.33	0	194.33	194.33	0.00	0.00
				6	214.18	229.85	58.27	104.31
				12	238.30	245.77	129.11	151.02
				18	246.13	256.21	152.09	181.67
				24	253.23	256.77	172.93	183.34
				30	256.77	256.77	183.34	183.34
				36	256.77	256.77	183.34	183.34
	III	232.40	192.40	0	192.40	192.40	0.00	0.00
				6	210.75	222.90	50.99	84.74
				12	223.66	232.40	86.87	111.14
				18	232.40	232.40	111.14	111.14
				24	232.40	232.40	111.14	111.14
				30	232.40	232.40	111.14	111.14
				36	232.40	232.40	111.14	111.14
4	I	257.88	187.11	0	187.11	187.11	0.00	0.00
				6	214.42	226.26	66.17	94.84
				12	230.56	250.48	105.26	153.53
				18	250.71	257.50	154.08	170.53
				24	255.49	257.88	165.67	171.46
				30	257.88	257.88	171.46	171.46
				36	257.88	257.88	171.46	171.46
	II	274.35	190.03	0	190.03	190.03	0.00	0.00
				6	220.80	234.86	80.21	116.88
				12	240.33	264.17	131.14	193.30
				18	260.50	270.24	183.73	209.11
				24	269.60	274.35	207.44	219.82
				30	274.35	274.35	219.82	219.82
				36	274.35	274.35	219.82	219.82
	III	232.73	189.87	0	189.87	189.87	0.00	0.00
				6	212.25	224.14	58.10	88.97
				12	228.73	232.73	100.88	111.26
				18	232.73	232.73	111.26	111.26
				24	232.73	232.73	111.26	111.26
				30	232.73	232.73	111.26	111.26
				36	232.73	232.73	111.26	111.26

Table 3 Efficiency loss (EL) values for different delay lengths, for a 4-arm MAMS with different values of J and unequally spaced interims, assuming uniform and linear recruitment. Here, the sample sizes are obtained assuming conjunctive power restrictions and a true global alternative,i.e. all treatments are effective. Here I, II and III represents the second row of table 1. The EL values are computed based on the equivalent single stage sample size of 370.14.

J	Spacing	n_{max}	ESS	Delay	ESS_{delay} (Uniform)	ESS_{delay} (Linear)	$EL_{Uniform}$	EL_{Linear}
2	I	373.87	325.43	0	325.43	325.43	0.00	0.00
				6	331.85	336.04	14.45	23.89
				12	343.64	373.87	41.01	109.11
				18	373.87	373.87	109.11	109.11
				24	373.87	373.87	109.11	109.11
				30	373.87	373.87	109.11	109.11
				36	373.87	373.87	109.11	109.11
	II	385.56	342.81	0	342.81	342.81	0.00	0.00
				6	345.87	346.88	11.34	15.08
				12	349.30	360.50	24.04	65.51
				18	360.95	385.56	67.18	158.24
				24	385.56	385.56	158.24	158.24
				30	385.56	385.56	158.24	158.24
				36	385.56	385.56	158.24	158.24
	III	370.55	328.97	0	328.97	328.97	0.00	0.00
				6	348.91	370.55	48.81	101.79
				12	370.55	370.55	101.79	101.79
				18	370.55	370.55	101.79	101.79
				24	370.55	370.55	101.79	101.79
				30	370.55	370.55	101.79	101.79
				36	370.55	370.55	101.79	101.79
3	I	389.67	307.43	0	307.43	307.43	0.00	0.00
				6	332.05	364.63	39.27	91.23
				12	375.14	377.51	107.98	111.75
				18	377.69	389.67	112.05	131.15
				24	389.67	389.67	131.15	131.15
				30	389.67	389.67	131.15	131.15
				36	389.67	389.67	131.15	131.15
	II	411.47	312.83	0	312.83	312.83	0.00	0.00
				6	339.44	368.48	46.44	97.11
				12	382.99	396.14	122.43	145.38
				18	397.04	410.51	146.95	170.45
				24	405.46	411.47	161.63	172.14
				30	411.47	411.47	172.14	172.14
				36	411.47	411.47	172.14	172.14
	III	375.19	309.16	0	309.16	309.16	0.00	0.00
				6	338.76	358.98	48.55	81.70
				12	361.27	375.19	85.46	108.28
				18	375.19	375.19	108.28	108.28
				24	375.19	375.19	108.28	108.28
				30	375.19	375.19	108.28	108.28

...Table 3 continued from previous page

				36	375.19	375.19	108.28	108.28
4	I	412.96	300.67	0	300.67	300.67	0.00	0.00
				6	341.36	358.88	58.71	84.00
				12	369.15	403.33	98.81	148.12
				18	403.85	412.35	148.87	161.14
				24	409.14	412.96	156.51	162.02
				30	412.96	412.96	162.02	162.02
				36	412.96	412.96	162.02	162.02
	II	439.74	306.31	0	306.31	306.31	0.00	0.00
				6	352.20	372.94	72.08	104.65
				12	384.08	425.75	122.16	187.59
				18	418.99	433.04	176.98	199.04
				24	432.00	439.74	197.41	209.57
				30	439.74	439.74	209.57	209.57
				36	439.74	439.74	209.57	209.57
	III	375.67	304.68	0	304.68	304.68	0.00	0.00
				6	337.99	361.01	51.01	86.27
				12	370.00	375.67	100.04	108.73
				18	375.67	375.67	108.73	108.73
				24	375.67	375.67	108.73	108.73
				30	375.67	375.67	108.73	108.73
				36	375.67	375.67	108.73	108.73

Table 4 Efficiency loss (EL) values for different delay lengths, for a 4-arm MAMS with different values of J and unequally spaced interims, assuming uniform and linear recruitment. Here, the sample sizes are obtained assuming conjunctive power restrictions and a true global alternative,i.e. all treatments are effective. Here I, II and III represents the third row of table 1. The EL values are computed based on the equivalent single stage sample size of 521.88.

J	Spacing	n_{max}	ESS	Delay	ESS_{delay} (Uniform)	ESS_{delay} (Linear)	$EL_{Uniform}$	EL_{Linear}
2	I	526.35	456.61	0	456.61	456.61	0.00	0.00
				6	463.24	470.64	10.11	21.39
				12	481.14	526.35	37.40	106.32
				18	526.35	526.35	106.32	106.32
				24	526.35	526.35	106.32	106.32
				30	526.35	526.35	106.32	106.32
				36	526.35	526.35	106.32	106.32
	II	540.53	481.58	0	481.58	481.58	0.00	0.00
				6	484.25	485.13	6.57	8.73
				12	489.45	504.80	19.36	57.15
				18	505.12	540.53	57.94	145.10
				24	540.53	540.53	145.10	145.10
				30	540.53	540.53	145.10	145.10
				36	540.53	540.53	145.10	145.10
	III	522.33	461.21	0	461.21	461.21	0.00	0.00
				6	489.74	522.33	46.78	100.20
				12	522.33	522.33	100.20	100.20

…Table 4 continued from previous page

				18	522.33	522.33	100.20	100.20
				24	522.33	522.33	100.20	100.20
				30	522.33	522.33	100.20	100.20
				36	522.33	522.33	100.20	100.20
3	I	546.27	429.29	0	429.29	429.29	0.00	0.00
				6	464.21	512.19	37.73	89.59
				12	527.68	530.66	106.33	109.55
				18	531.00	546.27	109.91	126.41
				24	546.27	546.27	126.41	126.41
				30	546.27	546.27	126.41	126.41
				36	546.27	546.27	126.41	126.41
	II	576.20	437.04	0	437.04	437.04	0.00	0.00
				6	472.15	516.94	41.41	94.23
				12	538.14	556.61	119.25	141.02
				18	557.93	574.79	142.58	162.47
				24	567.43	576.20	153.79	164.13
				30	576.20	576.20	164.13	164.13
				36	576.20	576.20	164.13	164.13
	III	527.60	432.09	0	432.09	432.09	0.00	0.00
				6	474.48	504.64	47.24	80.85
				12	507.56	527.60	84.11	106.44
				18	527.60	527.60	106.44	106.44
				24	527.60	527.60	106.44	106.44
				30	527.60	527.60	106.44	106.44
				36	527.60	527.60	106.44	106.44
4	I	578.46	419.61	0	419.61	419.61	0.00	0.00
				6	475.17	501.56	54.15	79.88
				12	515.50	566.75	93.47	143.42
				18	567.47	577.61	144.12	154.00
				24	573.11	578.46	149.62	154.84
				30	578.46	578.46	154.84	154.84
				36	578.46	578.46	154.84	154.84
	II	615.98	427.86	0	427.86	427.86	0.00	0.00
				6	490.90	520.94	66.82	98.66
				12	535.12	599.16	113.69	181.57
				18	587.75	606.48	169.47	189.33
				24	605.00	615.98	187.76	199.40
				30	615.98	615.98	199.40	199.40
				36	615.98	615.98	199.40	199.40
	III	528.67	425.77	0	425.77	425.77	0.00	0.00
				6	473.42	507.56	49.42	84.82
				12	521.16	528.67	98.92	106.70
				18	528.67	528.67	106.70	106.70
				24	528.67	528.67	106.70	106.70
				30	528.67	528.67	106.70	106.70
				36	528.67	528.67	106.70	106.70

4 | RESULTS FOR UNEQUALLY SPACED INTERIMS USING SYMMETRIC STOPPING BOUNDARIES

Figures 3 and 4 plot the EL values for unequally spaced MAMS for different delay lengths for uniform and linear recruitments respectively when we consider symmetric stopping boundaries, i.e. $f_j = -e_j, \forall j = 1, \cdots, J-1$. It can be observed from these figures that EL across different numbers of arms (K) remains similar.

The findings from this study aligns with our previous study on group sequential designs, i.e., if the first and subsequent interims are pushed towards the latter end of the trial, the EL tends to be larger. If the first interim analysis is conducted sooner than the first interim of an equally spaced design, the EL is the least. This is most evident in a 2-stage design as in this case the first (or the only interim in this case) is conducted at prominently different time points. For a 3 or 4 stage design the lines seem to overlap as the interim spacings are relatively close for different combinations (especially I and II). Also, another contributing factor to this increased EL is the ESS, as this takes the minimum value for an equally spaced design under no delay. Therefore, the relative loss inflates when compared to an equally spaced design vs an unequally spaced design. It might happen that the ESS_{delay} for an equally spaced design is still lower than the ESS_{delay} of an unequally spaced design. Therefore, careful inspection regarding the choice of the final design is necessary.

5 | RESULTS FOR THE TAILOR TRIAL ASSUMING THE GLOBAL NULL AND LEAST FAVOURABLE CONDITION

Tables 5,6 provide the efficiency losses for the TAILoR trial under a global null and the LFC.

□

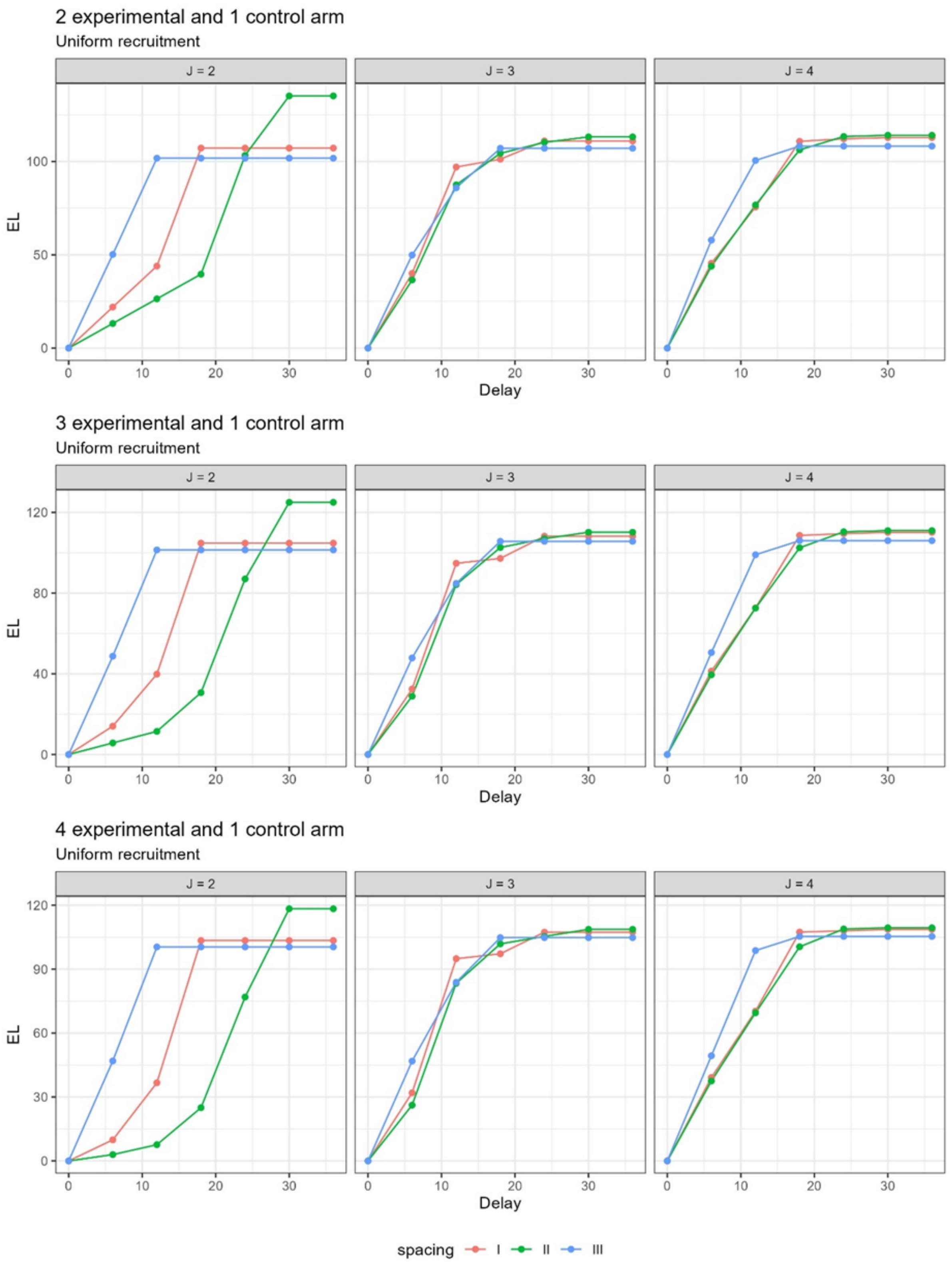

Figure 3 Efficiency loss (EL) for unequally spaced MAMS for different values of K and J assuming Uniform recruitment and symmetric stopping boundaries.

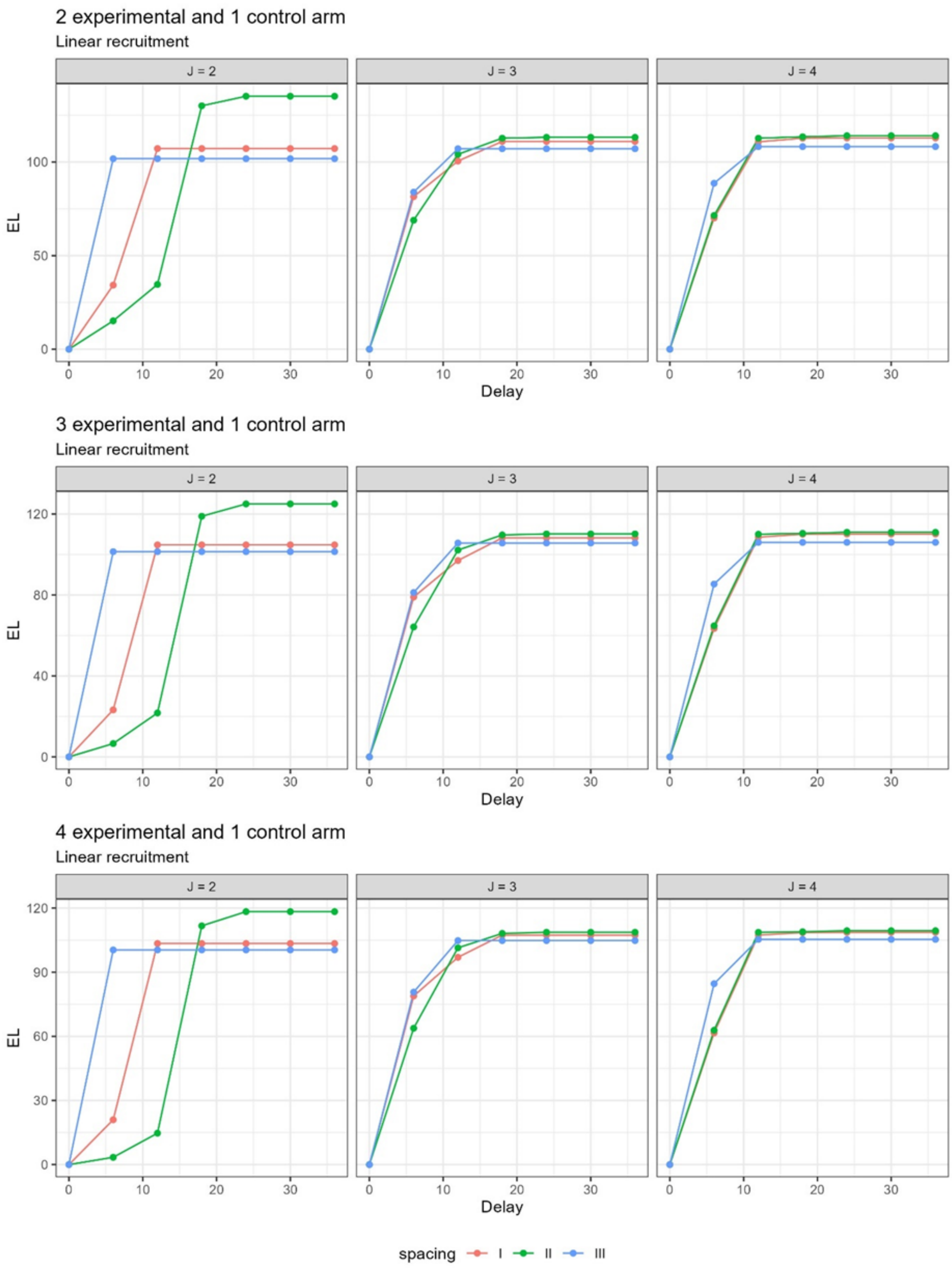

Figure 4 Efficiency loss (EL) for unequally spaced MAMS for different values of K and J assuming Linear recruitment and symmetric stopping boundaries.

Table 5 EL values for different recruitment rates and recruitment patterns for the TAILoR trial. The unit of time is months in the following table. The ESS given is derived assuming a true global null (no treatment is effective).

Stopping Boundary	t_{max}	m_0	No. of arms present in stage 2	$n_{delay}(\omega)$ (Uniform)	$n_{delay}(\omega)$ (Linear)	ESS	ESS_{delay} (Uniform)	ESS_{delay} (Linear)	$EL_{Uniform}$	EL_{Linear}
Original	29	6	0	69.52	112.15	304.43	308.26	314.15	7.58	19.26
			2	0.00	28.15					
	24	6	0	84.00	138.97	304.43	309.05	322.63	9.17	36.10
			2	0.00	54.97					
			3	0.00	12.97					
	20	6	0	113.70	184.16	304.43	312.10	336.00	15.00	61.77
			2	29.70	100.16					
			3	0.00	58.16					
OBF	29	6	0	73.81	119.08	278.64	297.10	315.89	24.21	48.87
			2	0.00	29.88					
	24	6	0	89.16	147.51	278.64	301.05	333.71	29.29	72.18
			2	0.00	58.35					
			3	0.00	13.76					
	20	6	0	107.06	178.44	278.64	309.75	356.60	40.90	102.32
			2	17.84	89.22					
			3	0.00	44.61					
Pocock	29	6	0	80.26	129.44	302.25	322.34	342.87	37.88	76.57
			2	0.00	32.49					
	24	6	0	96.96	160.41	302.25	326.55	362.34	45.89	113.59
			2	0.00	63.45					
			3	0.00	14.97					
	20	6	0	116.36	193.93	302.25	336.21	387.82	64.01	161.14
			2	19.39	96.96					
			3	0.00	48.48					
Triangular	29	6	0	78.07	125.96	293.90	313.53	333.46	31.91	64.49
			2	0.00	31.62					
	24	6	0	94.29	155.98	293.90	317.53	352.24	38.48	95.13
			2	0.00	61.70					
			3	0.00	14.55					
	20	6	0	113.11	188.52	293.90	326.95	376.97	53.97	135.68
			2	18.85	94.26					
			3	0.00	47.13					

Table 6 EL values for different recruitment rates and recruitment patterns for the TAILoR trial. The unit of time is months in the following table. The ESS given is derived assuming the least favourable condition (only one treatment is effective).

Stopping Boundary	t_{max}	m_0	No. of arms present in stage 2	$n_{delay}(\omega)$ (Uniform)	$n_{delay}(\omega)$ (Linear)	ESS	ESS_{delay} (Uniform)	ESS_{delay} (Linear)	$EL_{Uniform}$	EL_{Linear}
Original	29	6	0	69.52	112.15	274.76	278.85	292.69	5.07	22.25
			2	0.00	28.15					
	24	6	0	84.00	138.97	274.76	279.70	310.48	6.12	44.28
			2	0.00	54.97					
			3	0.00	12.97					
	20	6	0	100.80	168.00	274.76	287.45	336.00	15.89	76.71
			2	16.80	84.00					
			3	0.00	42.00					
OBF	29	6	0	73.81	119.08	300.64	302.48	314.72	3.37	25.78
			2	0.00	29.88					
	24	6	0	89.16	147.51	300.64	302.84	331.64	4.09	57.04
			2	0.00	58.35					
			3	0.00	13.76					
	20	6	0	107.06	178.44	300.64	310.15	356.85	17.23	103.67
			2	17.84	89.22					
			3	0.00	44.61					
Pocock	29	6	0	80.26	129.44	306.95	315.29	334.23	17.42	57.00
			2	0.00	32.49					
	24	6	0	96.96	160.41	306.95	316.96	356.56	20.74	102.34
			2	0.00	63.45					
			3	0.00	14.97					
	20	6	0	116.36	193.93	306.95	327.28	387.85	42.32	168.36
			2	19.39	96.96					
			3	0.00	48.48					
Triangular	29	6	0	78.07	125.96	301.69	308.60	326.13	12.94	45.73
			2	0.00	31.62					
	24	6	0	94.29	155.98	301.69	309.83	347.14	15.62	85.49
			2	0.00	61.70					
			3	0.00	14.55					
	20	6	0	113.11	188.52	293.90	319.42	377.04	33.47	141.09
			2	18.85	94.26					
			3	0.00	47.13					
